# Post-operative AICS status in completely resected lung cancer patients with pre-operative AICS abnormalities: predictive significance of disease recurrence

**DOI:** 10.1038/s41598-018-30685-2

**Published:** 2018-08-17

**Authors:** Takashi Anayama, Masahiko Higashiyama, Hiroshi Yamamoto, Shinya Kikuchi, Atsuko Ikeda, Jiro Okami, Toshiteru Tokunaga, Kentaro Hirohashi, Ryohei Miyazaki, Kazumasa Orihashi

**Affiliations:** 1 Division of Thoracic Surgery, Department of Surgery II, Kochi Medical School, Kochi University, Kochi, Japan; 2 Department of General Thoracic Surgery, Osaka International Cancer Institute, Osaka, Japan; 3 Research Institute for Bioscience Products & Fine Chemicals, Ajinomoto Co., Inc., Kanagawa, Japan; 4 Institute for Innovation, Ajinomoto Co., Inc., Kanagawa, Japan

## Abstract

The AminoIndex^TM^ Cancer Screening (AICS) system, a plasma-free amino acid (PFAA)-based multivariate discrimination index, is a blood screening test for lung cancer based on the comparison of PFAA concentrations between patients with lung cancer and healthy controls. Pre- and post-operative AICS values were compared among 72 patients who underwent curative resection for lung cancer. Post-operative changes in PFAA concentrations were also evaluated. AICS values were classified as rank A (0.0–4.9), B (5.0–7.9), or C (8.0–10.0). Rank B–C patients were evaluated for outcomes and post-operative changes in their AICS values. Twenty-three of the 44 pre-operative rank B–C patients experienced post-operative reductions in AICS rank. Only one patient experienced cancer recurrence. Post-operative changes in PFAA concentrations were associated with the risk of post-operative cancer recurrence (*p* = 0.001). Multivariate analysis revealed that the absence of a post-operative reduction in AICS rank independently predicted cancer recurrence (hazard ratio: 14.28; *p* = 0.012). The majority of patients had high pre-operative AICS values and exhibited a reduction in AICS rank after curative resection. However, the absence of a post-operative reduction in AICS rank was associated with cancer recurrence, suggesting that AICS rank may be a sensitive marker of post-operative recurrence.

## Introduction

Lung cancer is a leading cause of global morbidity and mortality, with approximately 14 million new cases in 2012^1^. Early detection and monitoring are important for improving treatment outcomes. Several serological markers (e.g., carcinoembryonic antigen [CEA] for adenocarcinoma and cytokeratin 19 fragment [CYFRA] for squamous cell carcinoma) have been used clinically for diagnostic and prognostic prediction and monitoring disease progression, recurrence, and response to treatment^2^. However, these tumour markers are not always elevated in the early stages of cancer development^3^. Therefore, the screening, diagnosis, and treatment of non-small-cell lung cancer could theoretically be improved by using a sensitive biomarker that can identify lung cancer and predict recurrence at an early stage.

Plasma-free amino acid (PFAA) concentrations are usually controlled at a constant ratio by homeostatic mechanisms. However, the balance of PFAA concentrations can vary in different diseases, including various cancers, liver or renal failure, and Alzheimer’s disease^4–6^. Therefore, we developed the AminoIndex^TM^ technology, which scores health conditions and the possibility of disease using multivariate analysis with PFAA concentrations included as a variable^7,8^. By comparing and analysing the ratios of 19 genetically encoded PFAAs in plasma samples derived from approximately 2,500 patients with cancer and approximately 15,000 healthy controls, we found that the PFAA concentration ratio was altered in 7 types of cancer, including lung cancer^9–16^. These findings allowed us to develop and clinically implement the AminoIndex™ Cancer Screening (AICS) system as an early screening test^10^. The lung-specific AICS (AICS (lung)) system identifies lung cancer based on the plasma concentrations of six statistically significant amino acids (serine, glutamine, alanine, histidine, ornithine, and lysine) and has been used to screen for lung cancer in Japan^10,11^. Based on the PFAA profile, the probability of lung cancer is expressed as AICS (lung) values of 0.0–10.0, which are categorised as rank A (0.0–4.9), B (5.0–7.9), and C (8.0–10.0)^10^. Higher AICS (lung) values are associated with an increased risk of lung cancer^10^.

Although AICS scores reflect PFAA concentrations, it is unclear whether these changes are related to the cancer itself or the underlying cause. The causal relationship between lung cancer and changes in PFAA concentrations has yet to be elucidated. For instance, if a high pre-operative AICS value is reduced to a lower AICS value after curative resection, it is possible that the index might reflect the presence of lung cancer and be a useful screening marker. Therefore, the present study aimed to evaluate post-operative changes in AICS (lung) values among patients who underwent curative resection for lung cancer, as well as the association of these changes with post-operative cancer recurrence. Our findings suggest that the pre- and post-operative PFAA profile may be a useful screening marker for lung cancer.

## Results

### Patient characteristics

Patient characteristics according to pre-operative AICS (lung) rank are shown in Table 1. The AICS (lung) rank was rank A in 28 patients (38.9%), rank B in 15 patients (20.8%), and rank C in 29 patients (40.3%). Forty-four patients (61.1%) had high pre-operative AICS (lung) values (rank B–C). No significant differences in rank were observed according to age, sex, pathological stage (pStage), tissue type, or treatment. Pre-operative AICS (lung) rank was associated with the degree of cancer differentiation, with rank C more frequently observed among patients with poorly differentiated cancer.

**Table 1:** Patient demographics.

Characteristic	Pre-operative AICS (lung)				*p*-value^*^
	All	Rank A	Rank B	Rank C	
	(n = 72)	(n = 28)	(n = 15)	(n = 29)	
Age (years)					0.314
Median (IQR)	64 (58–72)	59 (55–72)	65 (61–74)	64 (58–72)	
Range	34–79	34–77	35–78	44–79	
Sex, n (%)					0.822
Male	41 (56.9)	15 (53.6)	8 (53.3)	18 (62.1)	
Female	31 (43.1)	13 (46.4)	7 (46.7)	11 (37.9)	
pStage, n (%)					0.070
0	4 (5.6)	3 (10.7)	0 (0.0)	1 (3.4)	
I	47 (65.3)	22 (78.6)	11 (73.3)	14 (48.3)	
II	10 (13.9)	1 (3.6)	3 (20.0)	6 (20.7)	
III	10 (13.9)	2 (7.1)	1 (6.7)	7 (24.1)	
IV	1 (1.4)	0 (0.0)	0 (0.0)	1 (3.4)	
Histological type, n (%)					0.112
ADC	57 (79.2)	23 (82.1)	13 (86.7)	21 (72.4)	
SqCC	11 (15.3)	5 (17.9)	0 (0.0)	6 (20.7)	
Other	4 (5.6)	0 (0.0)	2 (13.3)	2 (6.9)	
Differentiation, n (%)					0.005
Well	25 (34.7)	16 (57.1)	5 (33.3)	4 (13.8)	
Moderate	35 (48.6)	10 (35.7)	9 (60.0)	16 (55.2)	
Poor	10 (13.9)	1 (3.6)	1 (6.7)	8 (27.6)	
Unknown	2 (2.8)	1 (3.6)	0 (0.0)	1 (3.4)	
Treatment, n (%)					0.817
S	62 (86.1)	25 (89.3)	13 (86.7)	24 (82.8)	
S + ACT	8 (11.1)	2 (7.1)	2 (13.3)	4 (13.8)	
S + ART	1 (1.4)	1 (3.6)	0 (0.0)	0 (0.0)	
S + ACT/ART	1 (1.4)	0 (0.0)	0 (0.0)	1 (3.4)	

^*^p-values calculated using the Kruskal-Wallis test for comparisons of medians, or Fisher’s exact test for comparisons of rank.

Abbreviations: ACT, adjuvant chemotherapy; ADC, adenocarcinoma; AICS (lung), lung-specific AminoIndex^TM^ Cancer Screening; ART, adjuvant radiotherapy; IQR, interquartile range; p-Stage, pathological stage; S, surgery; SqCC, squamous cell carcinoma.

### Pre-operative positive ratios

Pre-operative positive ratios of CEA, CYFRA, and AICS (lung) are shown in Table 2. Among 72 patients, 44 had an AICS (lung) rank of B–C. The sensitivity of AICS (lung) rank B–C, CEA, and CYFRA was 61.1%, 15.9%, and 5.3%, respectively. CEA was considerably higher in adenocarcinomas and CYFRA was considerably higher in squamous cell carcinomas.

**Table 2:** Pre-operative serum CEA, CYFRA, and AICS (lung) rank in patients with non-small-cell lung cancer.

	CEA (n = 69) high/normal (sensitivity, %)	CYFRA (n = 57) high/normal (sensitivity, %)	AICS (lung) (n = 72) rank C/B/A (sensitivity, %)*
ALL	11/58 (15.9)	3/54 (5.3)	29/15/28 (61.1)
ADC	9/46 (16.4)	2/42 (4.5)	21/13/23 (59.6)
SqCC	1/9 (10.0)	1/9 (10.0)	6/0/5 (54.5)
Other	1/3 (25.0)	0/3 (0.0)	2/2/0 (100.0)

^*^B-C *vs*. A.

Abbreviations: ADC, adenocarcinoma; AICS (lung), lung-specific AminoIndex^TM^ Cancer Screening; CEA, carcinoembryonic antigen; CYFRA, cytokeratin 19 fragment; SqCC, squamous cell carcinoma.

### Comparison of AICS (lung) rank before and after curative resection

**Figure 1:**
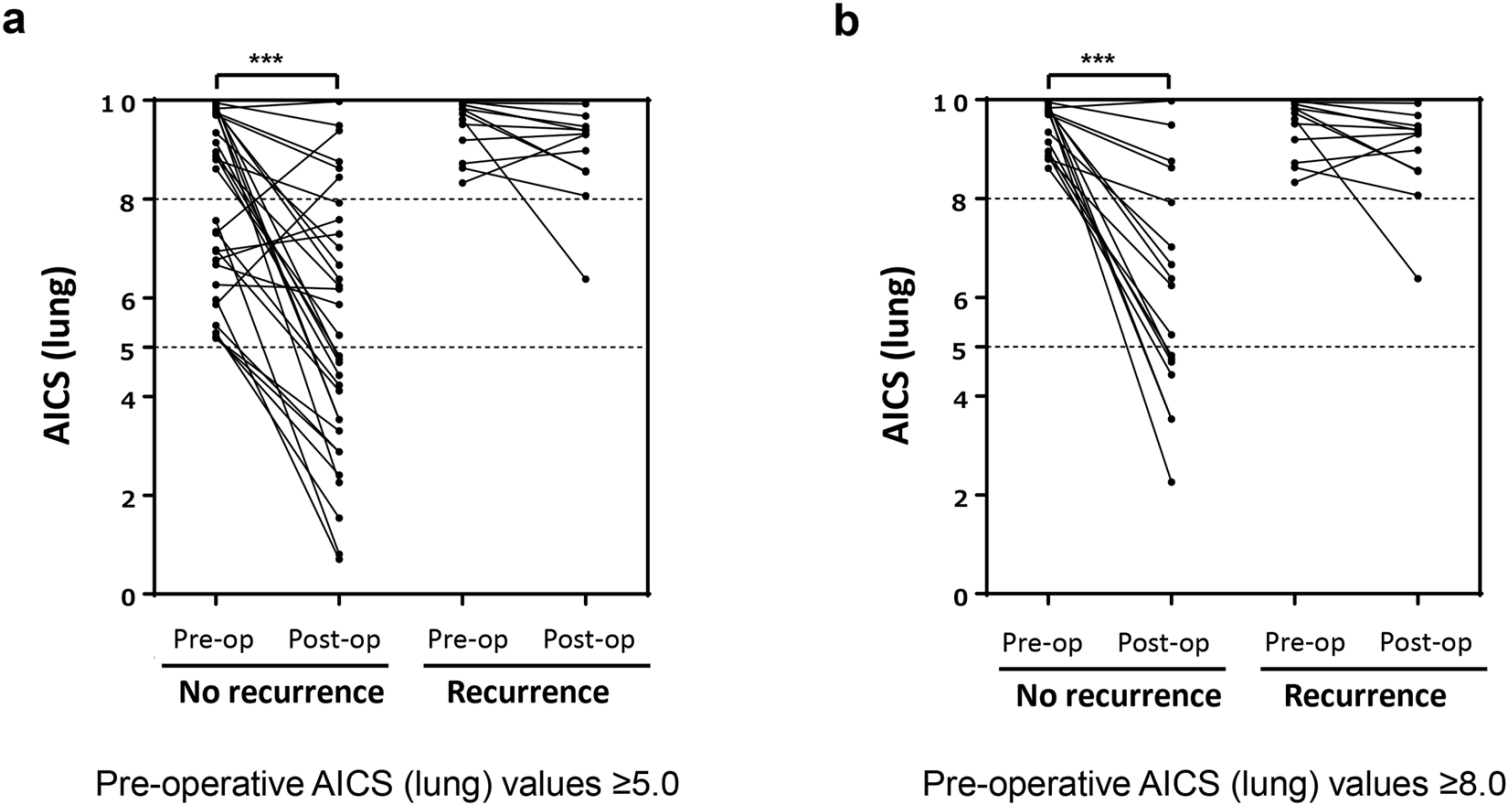
Post-operative changes in AICS (lung) values. Paired pre- and post-operative AICS (lung) values are shown for patients with a pre-operative AICS (lung) rank of (**a**) B and (**b**) C. The dotted line shows that a cut-off AICS (lung) value of 5.0 produces a specificity of 80.0%. Abbreviations: AICS (lung), lung-specific AminoIndex^TM^ Cancer Screening; pre-op., pre-operative; post-op., post-operative. ^***^Wilcoxon signed-rank, *p* < 0.001.

Post-operative changes in AICS (lung) rank were examined in 44 patients with a pre-operative AICS (lung) rank of B-C. After curative resection, the AICS (lung) rank was reduced to rank A–B in 23 patients (52.3%). Changes in AICS (lung) rank after curative resection according to whether the patient experienced recurrence or not are shown in Fig. 1a,b. Among 23 patients with a post-operative reduction in AICS (lung) rank, only one (4.3%) experienced post-operative recurrence. Among 21 patients with no post-operative reduction in AICS (lung) rank, 11 (52.4%) experienced post-operative recurrence.

Among 29 patients with a pre-operative AICS (lung) rank of C, 14 (48.3%) experienced a post-operative reduction in rank, with only one patient (7.1%) experiencing recurrence. In contrast, 11 (73.3%) of 15 patients without a post-operative reduction in AICS (lung) rank experienced recurrence. The absence of a post-operative reduction in AICS (lung) rank was associated with cancer recurrence (Table 3).

**Table 3:** Changes in AICS (lung) rank after curative resection among patients with a pre-operative AICS (lung) rank of B-C.

	Post-operative AICS (lung) rank		
	Reduction	No reduction	*p*-value^*^
Pre-operative AICS (lung) rank of B-C (n = 44)			
No recurrence	22 (95.7)	10 (47.6)	0.001
Recurrence	1 (4.3)	11 (52.4)	
Pre-operative AICS (lung) rank of C (n = 29)			
No recurrence	13 (92.9)	4 (26.7)	0.002
Recurrence	1 (7.1)	11 (73.3)	

^*^p-values calculated using Fisher’s exact test, n (%).

Abbreviations: AICS (lung), lung-specific AminoIndex^TM^ Cancer Screening.

Univariate and multivariate analysis of six factors (age, sex, pStage, histological type, cancer differentiation, and a post-operative reduction in AICS (lung) rank) revealed that the absence of a post-operative reduction in AICS (lung) rank (hazard ratio: 14.28; *p* = 0.012) and pStage II–IV disease (hazard ratio: 4.58; *p* = 0.024) were independent predictors of cancer recurrence (Table 4).

**Table 4:** Univariate and multivariate analysis.

Variables	Univariate analysis			Multivariate analysis		
	HR	95.0% CI	*p*-value	HR	95.0% CI	*p*-value
Age (years)	1.05	0.98–1.13	0.206	—	—	—
Sex (female *vs*. male)	0.98	0.31–3.09	0.974	—	—	—
pStage (II–IV *vs*. 0–I)	5.96	1.61–22.13	**0**.**008**	4.58	1.22–17.15	**0**.**024**
Histological type (ADC *vs*. Other)	0.49	0.15–1.63	0.244	—	—	—
Differentiation (poor/well *vs*. mod.)	1.57	0.41–5.97	0.511	—	—	—
CEA (≥5.0 *vs*. < 5.0 ng/mL)	1.92	0.57–6.41	0.292	—	—	—
Post-operative AICS (lung) rank (no reduction *vs*. reduction)	17.36	1.91–24.14	**0**.**007**	14.28	1.81–112.4	**0**.**012**

Abbreviations: ADC, adenocarcinoma; AICS (lung), lung-specific AminoIndex^TM^ Cancer Screening; CEA, carcinoembryonic antigen; CI, confidence interval; HR, hazard ratio; mod., moderate; pStage, pathological Stage.

### Changes in PFAA concentrations after curative resection

**Figure 2:**
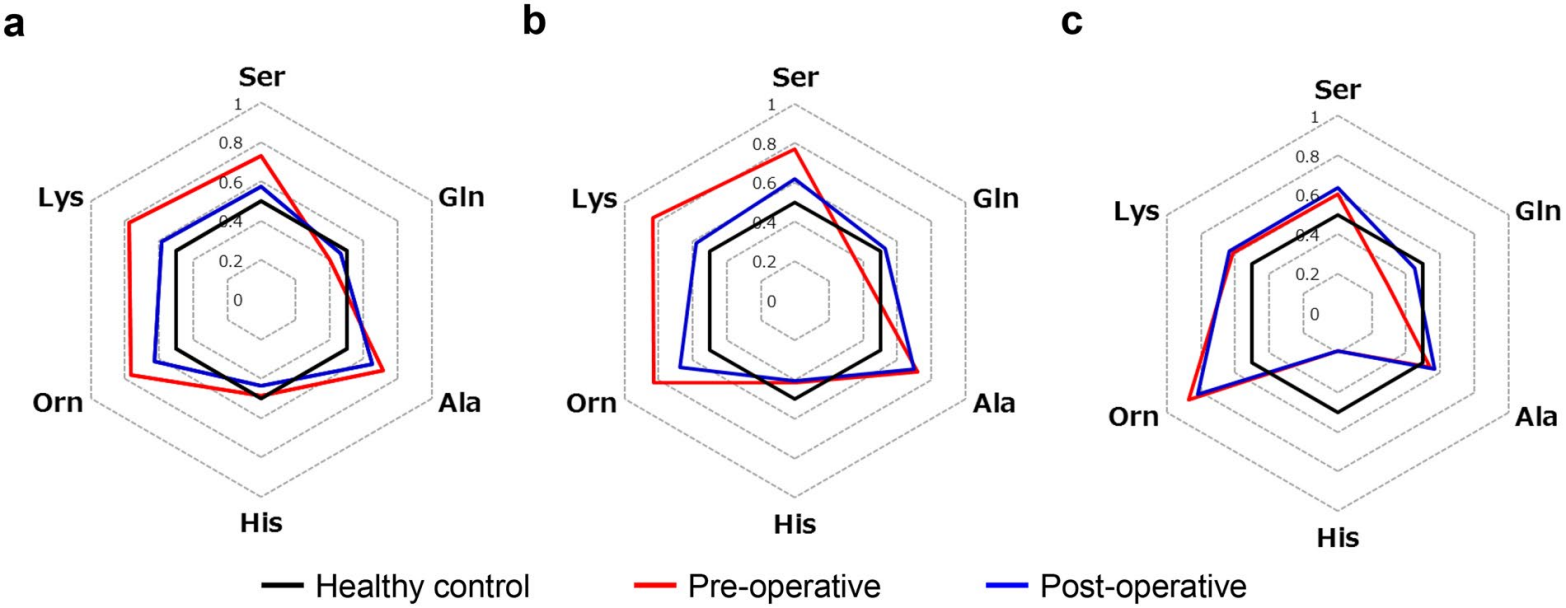
Radar charts of AICS (lung) amino acid concentrations among patients with a pre-operative AICS (lung) rank of B-C. Receiver operating characteristic curve analysis was performed for six amino acids. Areas under the receiver operating characteristic curves were used to determine whether each amino acid could discriminate between patients with lung cancer and healthy controls. (**a**) Patients with a pre-operative AICS (lung) rank of B–C who did not experience recurrence. (**b**) Patients with a pre-operative AICS (lung) rank of C who did not experience recurrence. (**c**) Patients with a pre-operative AICS (lung) rank of B–C who did experience recurrence. Only minor changes in post-operative amino acid concentrations were observed. Black bold lines indicate points where the area under the receiver operating characteristic curve was 0.5. Abbreviations: AICS (lung), lung-specific AminoIndex^TM^ Cancer Screening.

Pre- and post-operative plasma concentrations of serine, glutamine, alanine, histidine, ornithine, and lysine are shown in Fig. 2a–c. Patients who did not experience recurrence had similar post-operative PFAA concentrations compared to healthy controls. Patients who experienced recurrence did not have any noticeable change in post-operative PFAA concentrations compared to pre-operative PFAA concentrations.

### Disease-free survival by post-operative AICS (lung) rank status

**Figure 3:**
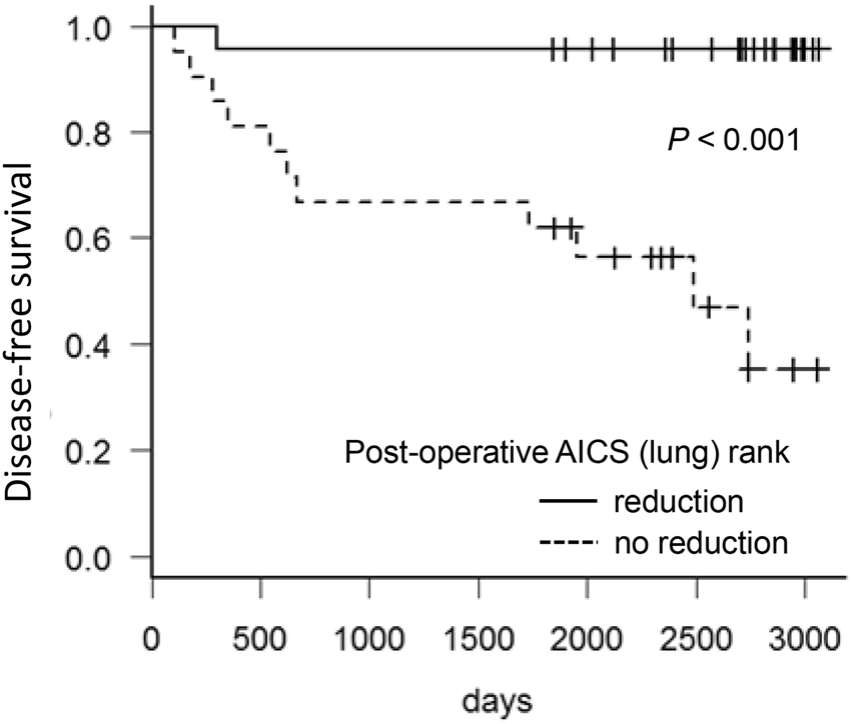
Disease-free survival of patients with a pre-operative AICS (lung) rank of B-C. Kaplan-Meier survival estimates of patients with or without AICS (lung) rank reduction. A log-rank test was used to test the significance, and censored patients are indicated by crosses. Abbreviations: AICS (lung), lung-specific AminoIndex^TM^ Cancer Screening.

For the 44 patients who were AICS (lung) rank B-C before surgery, the disease-free survival (DFS) was compared using Kaplan-Meier survival curves (Fig. 3). The group that maintained rank B-C after surgery had significantly worse DFS compared to the group with reduced AICS (lung) rank after surgery (*p* < 0.001).

## Discussion

In this study, we evaluated post-operative changes in AICS (lung) rank among patients who underwent curative resection for lung cancer. A pre-operative AICS (lung) rank of B-C was observed in 44 (61.1%) of 72 patients. A high rank was more frequently observed among patients with poorly differentiated cancer. In this context, lung cancer has diverse histological types and subtypes, with well-differentiated adenocarcinomas exhibiting non-invasive slow proliferation with lepidic growth that does not destroy the alveolar structure. Thus, recurrence is rare if well-differentiated adenocarcinomas are completely resected. However, poorly differentiated lung cancer is highly malignant and is associated with a poor prognosis^17,18^. Therefore, the high AICS (lung) rank among patients with poorly differentiated lung cancer may reflect its biological malignancy, although large-scale studies are needed to clarify the relationship between histological classification and AICS (lung) rank.

Surgical resection was associated with a reduction in post-operative AICS (lung) rank (C to A–B or B to A) in 23 (52.3%) of the 44 patients with a pre-operative AICS (lung) rank of B–C. Furthermore, 22 (95.7%) of the 23 patients experienced disease-free survival after curative resection, while 11 (52.4%) of the 21 patients with no post-operative change in AICS (lung) rank experienced post-operative recurrence. There was also a significant difference in DFS, as shown in Kaplan-Meier survival curve analysis based on post-operative changes in AICS (lung) rank. Moreover, the multivariate analysis revealed that the absence of a post-operative reduction in AICS (lung) rank and pStage II–IV disease were independent predictors of cancer recurrence after curative resection. It is possible that post-operative AICS (lung) rank accurately reflects the presence of residual lung cancer cells that were not detected during resection. Therefore, AICS (lung) rank may represent a sensitive tumour marker that can predict post-operative recurrence.

Of the six amino acids that are included in the AICS (lung) formula^10,11^, the concentrations of serine, glutamine, ornithine, and lysine in patients without post-operative recurrence were similar to those of healthy controls. Thus, it appears that these amino acids reflect the presence of residual cancer cells. It has recently been suggested that cancer cells indirectly affect the amino acid metabolism of distant organs and alter the PFAA profile^19^. However, there was no significant change in the post-operative concentration of alanine compared to its pre-operative concentration (Fig. 2a–c). Thus, although alanine is included in the AICS (lung) system to differentiate between patients with cancer and healthy controls, it is possible that the concentration of alanine may reflect predisposing background factors, such as the patient’s constitution and lifestyle, rather than the presence or absence of lung cancer. Given the uncertainty surrounding the mechanism by which cancer causes changes in the PFAA profile, further studies are needed to provide a more detailed analysis of the pre- and post-operative concentrations of each amino acid.

There is a limitation to this study. The 10 patients who had a pre-operative AICS (lung) rank of B–C and no post-operative reduction in AICS (lung) rank did not experience cancer recurrence during follow-up. However, long-term follow-up may be necessary to determine whether a high post-operative AICS (lung) rank in these cases was a false positive or whether recurrent lesions will emerge in the future.

A high pre-operative AICS (lung) rank and the absence of a post-operative reduction in AICS (lung) rank are risk factors for recurrence. AICS (lung) rank may be a sensitive marker of post-operative recurrence in patients with lung cancer.

## Methods

### Ethical considerations

This prospective observational study was performed in accordance with the Declaration of Helsinki, and the study protocol was approved by the Ethics Committees of the Osaka International Cancer Institute (Osaka, Japan) (formerly the Osaka Medical Center for Cancer and Cardiovascular Diseases) (ERB-000486) and the Kochi University School of Medicine (Kochi, Japan) (ERB-000137). All participants gave their written informed consent for inclusion in this study. All data were analysed anonymously.

### Patient recruitment

Patients who underwent curative resection for primary lung cancer at the Osaka International Cancer Institute (Osaka, Japan) or Kochi University School of Medicine (Kochi, Japan) between January 2007 and December 2010 were enrolled. Patients underwent pre-operative blood sampling within 1 week prior to surgery and post-operative blood sampling 0.7–5.5 years after surgery. The median follow-up time was 7.1 (range, 1.7–8.7) years. Patients were excluded if they had a history of other malignancies. Tumours were classified according to the American Joint Committee on Cancer and Union for International Cancer Control Lung Cancer Stage Classification (8^th^ edition)^20^. Histological classification and histological grading was performed according to the 8^th^ ^21^ and 7^th^ ^22^ editions of the TNM Classification of Malignant Tumors.

### Measurements of PFAA concentrations

Blood samples (5.0 mL) were collected from a forearm vein on the morning after an overnight fasting, using tubes containing ethylenediaminetetraacetic acid, and were immediately placed on ice. Plasma was prepared by centrifugation at 3,000 rpm for 15 minutes at 4 °C and was stored at −80 °C until analysis. Plasma samples were deproteinised using acetonitrile at a final concentration of 80.0%. PFAA concentrations were measured using high-performance liquid chromatography/electrospray ionisation tandem mass spectrometry with pre-column derivatisation, which have been described previously^23–25^.

### AICS (lung) values and ranks

AICS (lung) values for patients with lung cancer were classified as rank A, B, or C based on a previous report^10^. AICS (lung) detects lung cancer from plasma concentrations of serine, glutamine, alanine, histidine, ornithine, and lysine^10,14^. AICS (lung) values range between 0.0 and 10.0, with 5.0 producing 80.0% specificity and 8.0 producing 95.0% specificity. A high AICS (lung) value is associated with a greater probability of lung cancer. Rank A is considered to be normal (0.0–4.9), rank B is considered to be relatively high (5.0–7.9), and rank C is considered to be high (8.0–10.0).

### Statistical analyses

Statistical analyses were performed using Fisher’s exact test or the Kruskal-Wallis test, as appropriate. Post-operative changes in AICS (lung) rank were evaluated using the Wilcoxon signed-rank test. Receiver operating characteristic curve analyses were performed to determine the abilities of PFAA concentrations and the post-operative AICS (lung) rank to differentiate between patients with lung cancer and healthy controls^11^. The 95.0% confidence intervals for the areas under the receiver operating characteristic curves were estimated based on the method described by Hanley and McNeil^26^. Multivariate analysis was performed using Cox regression model. Results were presented as hazard ratios and 95.0% confidence intervals. Survival curves were drawn by the Kaplan-Meier method, and a statistical evaluation of the curves was performed using a log-rank test. All statistical analyses were conducted using GraphPad Prism (software version 6; GraphPad Software Inc., San Diego, CA, USA) and R software (version 3.2.2; The R Foundation for Statistical Computing, Vienna, Austria).

## Data Availability

The datasets generated during and/or analysed during the current study are not publicly available due to the nature of the ethical approval of this study but are available to readers who meet the criteria for access to confidential data from the Ethics Committee of Kochi University School of Medicine (Kochi, Japan) (im32@kochi-u.ac.jp).
